# Zinc, copper, and magnesium in premenstrual disorders: a narrative review

**DOI:** 10.1007/s43440-025-00791-w

**Published:** 2025-10-15

**Authors:** Anna Julia Krupa, Magdalena Zybała-Pawłowska, Michał Kania, Justyna Turek, Bernadeta Szewczyk, Andreas M. Grabrucker, Marcin Siwek

**Affiliations:** 1 Department of Affective Disorders, Jagiellonian University Medical College, Kopernika 21a, Kraków, 31-501 Poland; 2 Clinical Department of Adult, Child and Adolescent Psychiatry, University Hospital, Kopernika 21a, Kraków, 31-501 Poland; 3 Department of Neurobiology, Maj Institute of Pharmacology, Polish Academy of Sciences, Smętna 12, Kraków, 31-343 Poland; 4 Department of Biological Sciences, University of Limerick, Limerick, V94PH61 Ireland; 5 Bernal Institute, University of Limerick, Limerick, V94PH61 Ireland; 6 Health Research Institute (HRI), University of Limerick, Limerick, V94PH61 Ireland

**Keywords:** Premenstrual syndrome, Premenstrual dysphoric disorder, Zinc, Copper, Magnesium

## Abstract

Premenstrual disorders (PMD) are a prevalent health issue and often co-occur with mood disorders. The pathophysiology of PMD has not yet been thoroughly described. Two mechanisms appear to be crucial in PMD: (1) lower estrogen levels during the luteal phase, leading to a subsequent decrease in serotonin (5-HT) transmission, and (2) reduced sensitivity to allopregnanolone, resulting in an imbalance in γ-aminobutyric acid (GABA)/glutamate signaling and an increase in hypothalamic-pituitary-adrenal (HPA) activation. The roles of zinc (Zn), copper (Cu), and magnesium (Mg) in mood disorders are well-established, and they appear to be associated with PMD through similar pathways. Therefore, this narrative review provides background information on the roles of Zn, Cu, and Mg in mood regulation and discusses the impact of these trace elements on this process. The results presented, summarizing data from studies: (1) exploring the associations between Zn, Cu, and Mg levels and PMD, and (2) verifying the effects of Zn, Cu, and Mg supplementation on PMD symptoms. Finally, the caveats of current PMD research and the implications of the available data for everyday clinical practice are discussed.

**Clinical trial number**: Not applicable.

## Introduction

Premenstrual symptoms refer to both psychological and somatic distress that present in the luteal phase of the menstrual cycle [1]. Epidemiologically, such disturbances in their mild form seem to be highly prevalent across high-, middle-, and low-income countries, affecting between 80 and 90% of the female population [1, 2]. Nevertheless, they usually do not significantly impair women’s functioning [3]. More severe cases are diagnosed as premenstrual syndrome (PMS) according to gynecological classifications or premenstrual dysphoric disorder (PMDD) in psychiatric nosology (Table 1) [1]. Although categorized into nonhomogeneous diagnostic categories, PMS (a milder form) and PMDD (a more severe presentation) appear to represent a spectrum of the same pathology; therefore, an umbrella term of premenstrual disorders (PMD) has been proposed for these phenomena [4]. It should be noted that heterogeneous diagnostic constructs are used in the literature: premenstrual syndrome (PMS) is defined based on gynecological classification, premenstrual dysphoric disorder (PMDD) based on psychiatric nosology, while older studies often used the historical term premenstrual tension syndrome (PMTS). These categories should not be considered equivalent.

**Table 1:** Diagnostic criteria for PMS according to the American college of obstetricians and gynecologists (ACOG), and PMDD according to DSM-5-TR [3]

PMS	PMDD
**Diagnostic Criteria**: Diagnosis requires ≥ 1 of the following affective or somatic symptoms during the 5 days before menses in each of the 3 previous cycles	**Diagnostic Criteria**: Symptoms must be present in most menstrual cycles over the past year, with ≥ 5 symptoms in the final week before menses. Symptoms must improve within a few days after onset of menses and become minimal or absent post-menses.
**Affective symptoms**: 1. Angry outbursts 2. Anxiety 3. Confusion 4. Depression 5. Irritability 6. Social withdrawal	**Affective symptoms**: At least one of the following must be present: 1. Marked affective lability. 2. Marked irritability or anger, or interpersonal conflicts. 3. Marked depressed mood, hopelessness, self-deprecating thoughts 4. Marked anxiety, tension, or feelings on edge
**Somatic symptoms**: Diagnosis includes ≥ 1 of the following physical symptoms: 1. Abdominal bloating 2. Breast tenderness or swelling 3. Headache 4. Joint or muscle pain 5. Swelling of extremities 6. Weight gain These symptoms must be relieved within 4 days after the onset of menses, and not recur until at least day 13 of the cycle.	**Additional Symptoms (to meet ≥ 5 total)**: At least one of the following must also be present: 1. Decreased interest in usual activities 2. Subjective difficulty in concentration 3. Lethargy, easy fatigability, or marked lack of energy 4. Marked change in appetite, including overeating or food cravings 5. Hypersomnia or insomnia 6. A sense of being overwhelmed or out of control 7. Physical symptoms such as: breast tenderness or swelling, joint or muscle pain, sensation of “bloating”, weight gain
The symptoms must occur reproducibly during at least two cycles of prospective recording.	Prospective daily ratings must confirm the presence of symptoms during at least two symptomatic cycles
Symptoms must cause identifiable dysfunction in social, academic, or occupational performance.	Symptoms must cause clinically significant distress or impairment in work, school, social activities, or interpersonal relationships.
Symptoms must be present without the influence of pharmacologic therapy, hormone ingestion use, drugs, or alcohol.	Symptoms must not be attributable to the physiologic effects of a substance
	The disturbance must not be an exacerbation of another mental disorder (e.g. major depressive disorder, panic disorder, dysthymia, or a personality disorder), although it may co-occur with these conditions.

^1^ PMDD – premenstrual dysphoric disorder, PMS – premenstrual syndrome

It is estimated that among women of reproductive age, PMS affects 20–43% [5, 6] while PMDD is present in 3.2–7.7% of the general population [7]. Historically, the significance of premenstrual symptoms was first acknowledged in the scientific field by Frank in 1931, who recognized not only the PMD symptoms but also the toll it took on his patients’ self-perception and their relationships [8]. Intriguingly, PMD researchers have long recognised the influence of nutritional deficiencies and micronutrient supplementation; however, no clear role for specific trace elements or consistent clinical implications has been established [1, 9–11]. PMD significantly impacts the overall health and well-being of women, contributing to higher levels of depression, anxiety, suicidal risk, impaired social and educational/work performance, and lower quality of life [7, 8, 12–15]. Moreover, PMD is often comorbid with mood disorders. It is estimated that 10% of women with PMDD have bipolar disorder, which poses a 7-fold higher risk compared to women without PMDD [16]. Furthermore, self-reported PMS is a predictor of a future depression diagnosis [17], and the odds ratio of PMDD is much higher in women with depression (compared to subjects with no history of depression) [18].

Zinc (Zn), copper (Cu), and magnesium (Mg) are essential trace elements that participate in various neurobiological pathways of high relevance to premenstrual disorders (PMD), including oxidative stress regulation, neurotransmitter modulation, inflammatory responses, and hypothalamic–pituitary–adrenal (HPA) axis function [19–21]. Zn supports serotonergic and GABAergic neurotransmission and has anti-inflammatory and antioxidant properties [22, 23]. Cu is essential for catecholamine metabolism, but its excess may contribute to oxidative damage and mood disorders [20]. Mg, in turn, modulates calcium influx into neurons and supports the synthesis of serotonin and GABA, which play a key role in emotion regulation and stress adaptation [21, 24]. These overlapping biological roles provide a compelling argument for investigating Zn, Cu, and Mg in the context of PMD pathophysiology and symptom treatment. Therefore, this narrative review aims to summarize the data on (1) PMD pathophysiology and how Zn, Cu, and Mg might influence it; (2) studies exploring the associations between Zn, Cu, and Mg levels with PMD; and (3) studies verifying the effects of Zn, Cu, and Mg supplementation on PMD symptoms. Zn, Cu, and Mg were selected due to their significant role in regulating mood, oxidative stress, and neurotransmission, which are key to understanding the pathophysiology of PMD [20, 25, 26].

## Pathophysiology of PMD

**Fig. 1:**
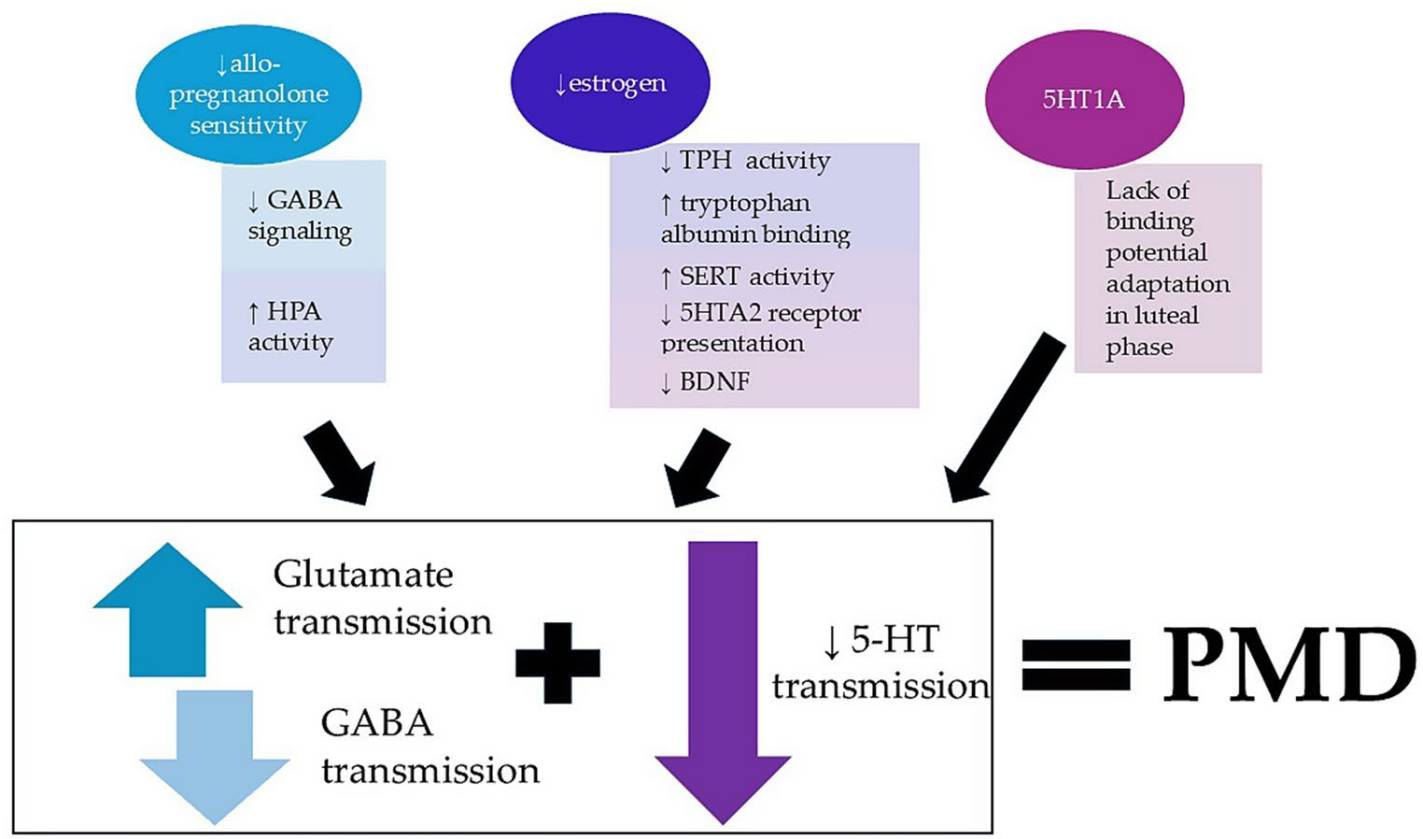
Pathophysiology of premenstrual disorders

A coherent understanding of PMD is still lacking, but current data points to the interplay between female sexual hormones and their impact on serotonin (5-HT) and γ-aminobutyric acid (GABA) neurotransmission [27] (Fig. 1). Firstly, PMD is only present in menstruating women; PMD begin after menarche, the symptoms are absent during pregnancy, and the condition resolves after menopause [4]. Secondly, the impact of hormones on PMD appears to be primarily due to individual differences in sensitivity to fluctuations in ovarian hormone levels during the luteal phase of the menstrual cycle. Studies have shown that PMD symptoms are suppressed by the administration of gonadotropin-releasing hormone (GnRH) agonists and recur upon estrogen and progesterone supplementation in physiological doses [28]. Additionally, the use of continuous oral contraception (levonorgestrel and ethinyl estradiol in fixed doses) alleviates PMD symptoms [29].

There appear to be two significant pathways through which hormonal evolution during the menstrual cycle translates into altered neurotransmission during the luteal phase in individuals with PMD. One pathway involves the decrease of estrogens and their impact on 5-HT signaling, impairments that are known to play a vital role in mood and anxiety disorders [30]. Estrogens promote serotonergic transmission by upregulating the expression of the rate-limiting enzyme tryptophan hydroxylase (TPH), thereby increasing tryptophan levels (which is necessary for 5-HT synthesis) and downregulating the expression of the serotonin transporter (SERT), resulting in increased availability of 5-HT in the synaptic cleft [31, 32]. Additionally, estrogens have been shown to increase the presentation of 5-HT2A receptors in brain regions related to mood, cognition, and reward, leading to desensitization of 5-HT1A autoreceptors and disinhibition of 5-HT neurons’ transmission [31, 33]. Interestingly, studies indicate that the 5-HT1A receptor binding potential changes depending on the menstrual cycle phase in healthy women, but this effect is less pronounced in individuals with PMDD [34]. Moreover, estrogens compete with tryptophan for albumin binding; therefore, higher serum levels of estrogens result in greater availability of free tryptophan. They also promote the release of brain-derived neurotrophic factor (BDNF), stimulating the development and plasticity of 5-HT neurons [32]. Indeed, estrogen replacement in postmenopausal women was shown to promote 5-HT transmission [35], and selective serotonin reuptake inhibitors (SSRIs) are known to be effective in alleviating PMD, even when taken only during the luteal phase of the menstrual cycle [36].

The other way hormonal fluctuations during the menstrual cycle impact neurotransmission is through allopregnanolone modulation of GABA_A_ receptors [37]. GABA is the primary inhibitory transmitter in the human central nervous system, and many anxiolytic drugs (benzodiazepines) enhance GABA transmission. In healthy women, the administration of allopregnanolone activates GABA_A_ receptors. It promotes sedation, while in women with PMDD, allopregnanolone induces sedation only during the follicular but not the luteal phase of the cycle [38]. Additionally, physiologically, allopregnanolone acts as a mitigator of the stress response by attenuating corticotropin release from the hypothalamus and provides negative feedback for the activated hypothalamus-pituitary-adrenal (HPA) axis. This mechanism is blunted in women with PMD, resulting in increased sensitivity to stress during the luteal phase [39, 40]. Interestingly, the effect of allopregnanolone on GABA transmission and the HPA axis in PMDD is normalized by SSRI treatment [39, 41].

Hormonal fluctuations during the menstrual cycle influence neurotransmission related to mood disorders. Estrogens alter serotonergic transmission by affecting TPH activity, increasing 5-HT availability, and modulating 5-HT2A receptor presentation. They also impact free tryptophan levels and modify BDNF release, which supports the plasticity of 5-HT neurons. Allopregnanolone affects GABA_A_ receptor activity. Additionally, allopregnanolone can moderate the stress response through the HPA axis.

5-HT - serotonin, BDNF - brain-derived neurotrophic factor, GABA - γ-aminobutyric acid, HPA - hypothalamic-pituitary-adrenal, SERT - serotonin transporter, TPH- tryptophan hydroxylase.

## Zinc

### Zinc impact on mood and PMD pathophysiology

**Fig. 2:**
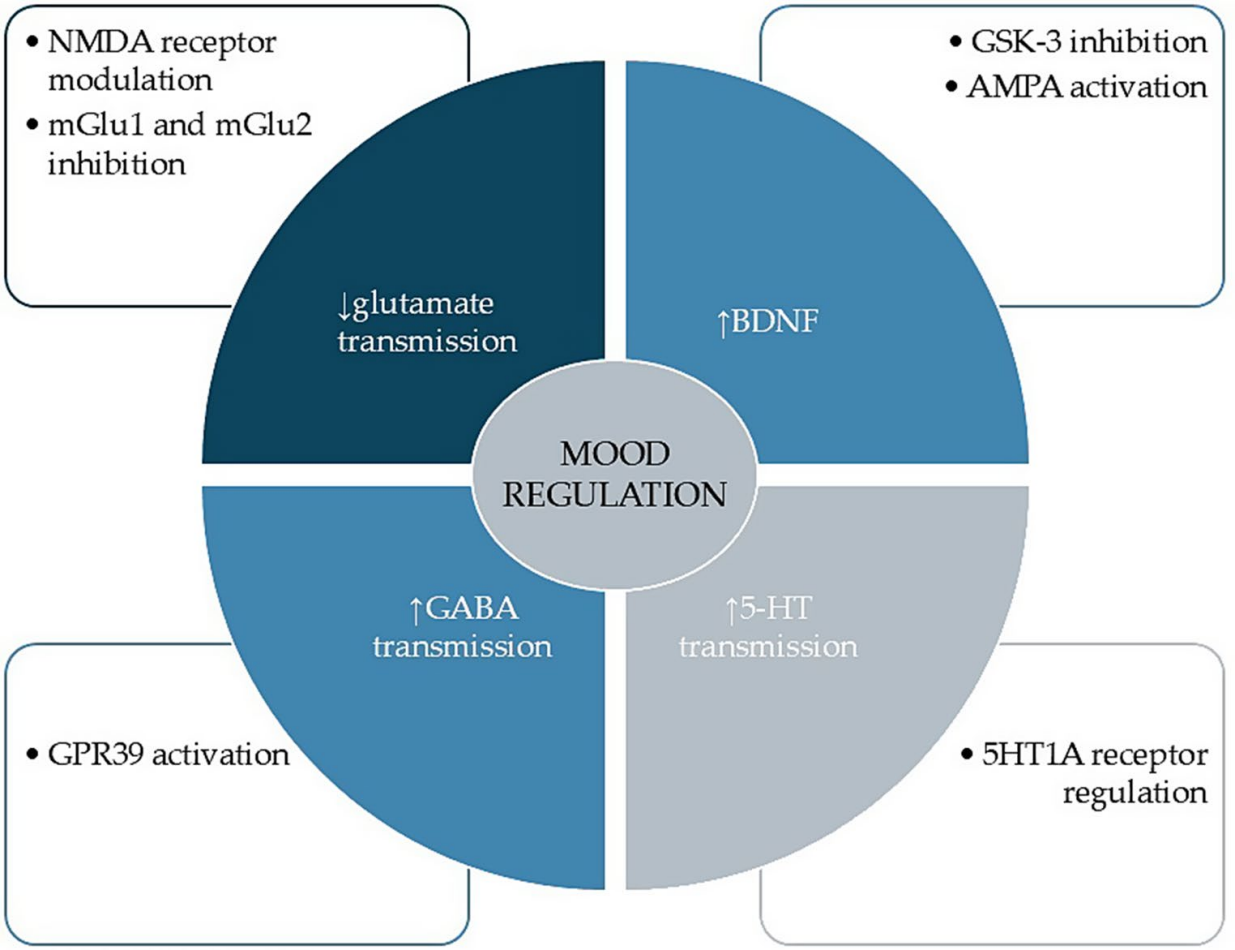
Zinc’s impact on mood and the pathophysiology of premenstrual disorders

The imbalance between excitatory (glutamate-based) and inhibitory (GABA-based) systems of neurotransmission in the CNS is central to mood disorders, and Zn plays a significant role in mood regulation by stabilizing it [19]. The impact of Zn on glutamate signaling is well described, indicating that Zn modulates and inhibits the N-methyl-D-aspartate (NMDA) receptors for glutamate [26]; it also inhibits group metabotropic glutamate receptors I (group I mGluRs) and group II (group II mGluRs) and activates ionotropic α-amino-3-hydroxy-5-methyl-4-isoxazolepropionic acid (AMPA) receptors, which are the mechanisms for novel antidepressant action, i.e., ketamine [42]. Moreover, Zn promotes neurotrophic transmission by inhibiting glycogen synthase kinase-3 (GSK-3) and disinhibiting the CREB/BDNF pathway [22]. Zn deficiency induces overactivity and upregulation of the NMDA receptors, increasing the risk of excitotoxicity [43], and it has been linked to the occurrence of depression [23]. The supplementation of Zn potentiates the antidepressant effect in treatment-resistant depression [44, 45]. However, our understanding of Zn’s impact on GABA signaling remains to be thoroughly elucidated. Zn acts as an inhibitor on some GABA_A_ receptor isoforms at high concentrations when applied exogenously. Still, physiologically, it is unlikely to inhibit GABA, and indeed, further studies have indicated that Zn promotes the amplitude of mossy fibre GABA Inhibitory Postsynaptic Currents (IPSCs) [46] and enhances GABA signaling in neocortical neurons [47]. Recently, it was reported that both Zn and the Zn-sensing receptor GPR39 induce antidepressant effects by modulating GABA [19]. Furthermore, animal studies suggest that zinc (Zn) enhances 5-HT transmission in the hippocampus by regulating the 5-HT1A receptor [48] (Fig. 2).

Zn stabilizes the excitatory (glutamate) and inhibitory (GABA) balance in the CNS, influencing mood disorders. It inhibits NMDA receptors and mGluR1/II, while enhancing AMPA receptors. Zn deficiency leads to NMDA receptor overactivity and depression, while supplementation boosts antidepressant effects. Zn’s effects on GABA signaling remain complex, but it enhances GABA transmission in specific contexts. Zn and the receptor GPR39 collectively induce antidepressant effects, with Zn additionally increasing 5-HT transmission via 5-HT1A receptor regulation.

5-HT- serotonin, AMPA- α-amino-3-hydroxy-5-methyl-4-isoxazole propionic acid, BDNF- brain-derived neurotrophic factor, GABA - γ-amino butyric acid, GPR39- Zn-sensitizing receptor, GSK-3- glycogen synthase kinase-3, mGluR1- metabotropic glutamate receptors group I, metabotropic glutamate receptors group I, mGluR2 - NMDA- N-methyl-D-aspartate.

### Serum zinc levels and PMD

Lower Zn levels are consistently found in people with bipolar disorder and depression [23, 45], and similar observations were made in PMD (Table 2). In a cross-sectional study of women with premenstrual tension syndrome (PMTS) compared to controls, a significantly lower Zn/Cu ratio (in the luteal phase) was observed in PMTS than in controls (the Zn/Cu ratio is used to depict Zn availability because Cu and Zn compete for intestinal absorption and serum protein binding sites). Additionally, plasma Zn was lower during the luteal phase than in the follicular phase of the cycle in PMTS, but not in healthy women [49]. Another cross-sectional study indicated that women with PMS present lower serum Zn and lower serum total antioxidant capacity (TAC), as measured in the luteal phase, than healthy females [50]. Further work revealed that serum Zn levels and TAC negatively correlate with PMS severity [51].

**Table 2:** Studies exploring the role of Zn, and Zn and Cu in PMD

Study design	Population	Outcomes	References
Cross-sectional study, measurement of serum Zn, Cu, Mg in the luteal phase (5 days before menses) and follicular phase (6 days after menses onset) Assessment of PMS with MDQ	40 women with PMTS and 20 HC	Lower mean plasma Zn and Zn/Cu ratio in PMTS women vs. HC in the luteal phase Similar mean plasma Cu in PMTS women vs. HC in the luteal and follicular phases	[49]
Cross-sectional study, measurement of serum Zn, TAC on the 21st day of the menstrual cycle Evaluation of PMS with a five-point Likert-type scale of 44 items Assessment of nutrient intake and physical activity via questionnaires and interviews	48 women with PMS and 62 HC	Lower mean plasma Zn and lower TAC in PMS women vs. HC	[50]
Case-control study, measurement of serum Zn, TAC in the 3rd week of the cycle Dietary intake questionnaire PMS was assessed with the Daily Symptom Rating Scale (DSR)	23 women with PMS and 25 HC	Lower mean plasma Zn and lower TAC in PMS women vs. HC Negative correlation between mean plasma PMS severity scores and serum Zn / TAC	[51]
Prospective, observation, measurement of serum Zn and Cu via regular blood sampling in 2–3 day intervals, for 3 full menstrual cycles PMS assessed with daily dairy and MDQ	13 women with PMS and 10 HC	Lower plasma Zn levels in the luteal vs. the follicular phase in PMS Lower plasma Zn levels and Zn/Cu ratio in PMS vs. HC in the luteal phase Higher plasma Cu levels in PMS vs. HC in the luteal phase	[52]
Cross-sectional study, measurement of serum levels of vitamin D, Ca, Mg, Zn, Fe, K, Na taken 2 weeks after menses, PMS assessed with PSST	115 women with PMS and 163 HC	No differences in serum Zn in PMS women vs. HC	[53]
Cross-sectional study, measurement of serum Zn, Mg, vitamin A, vitamin E, and activity of erythrocyte enzymes in the luteal phase (5 days before menses) and follicular phase (6 days after menses onset) PMTS, diagnosed by a family doctor or a gynecologist	38 women with PMS (12 reported significant mood cyclicity) and 23 HC	No significant differences in the assessed parameters in the studied groups	[54]
Experimental study, measurement of serum Zn level and sweet taste sensitivity in the luteal phase	7 women with PMS or HC	No significant differences in serum Zn or sweet taste sensitivity	[55]
Cross-sectional study, assessment of Ca, Mg, Zn, Cu, and 14 other elements in blood samples (lack of data on the time of sample collection) and hair samples	46 women with PMS and 50 HC	Lower Ca, Cu, chromium, and manganese in PMS vs. HC blood Higher Zn, Na, lead, arsenic, and germanium in PMS vs. HC blood	[56]
RCT, one group received 220 mg Zn sulfate (50 mg Zn)(*n* = 33), the other group received a placebo daily for 24 weeks PMS assessed with PSST (adolescent version), at baseline and after 1,3,6 months	69 women with PMS	Significant reduction of PMS symptoms and improved functioning in the group receiving Zn vs. placebo after 1,3, and 6 months of intervention	[57]
RCT one group received Zn gluconate (30 mg Zn)(*n* = 30), other group received placebo (*n* = 30) for 12 weeks PMS severity assessed with PSST, and blood collected for evaluations of TAC, hCRP, and BDNF at baseline and after 12 weeks	60 women with PMS	Significant reduction of PMS symptoms and an increase in TAC as well as BDNF in subjects receiving Zn vs. individuals receiving placebo after 12 weeks of intervention	[58]
10 years nested case-control study Mineral intake was assessed using food frequency questionnaires at the 1st, 5th, and 9th years of the study	1,057 women with PMS and 1,968 HC	Negative association between high Zn intake (≥ 25 mg/day) vs. none, which was marginally significant	[59]
RCT, one group received 220 mg Zn sulfate (50 mg Zn) (*n* = 71), the other group received a placebo (*n* = 71) from the 16th day to the 2nd day of the menstrual cycle, for 3 cycles PMS severity assessed with PSST, evaluations after 1,2, and 3 months	142 women with PMS	Significant reduction in the PMS severity and improvement of quality of life in the group receiving Zn vs. placebo after 3 months	[60]
A 3-day food record during the perimenstrual phase was conducted to assess energy intake, diet composition, and consumption of macro- and micronutrients PMS was evaluated with the Daily Record of Severity of Problems	16 women with PMS and 14 HC	Higher Cu intake in PMS women vs. HC Similar (suboptimal) Zn intake in PMS women and HC	[70]

BDNF – brain-derived neurotrophic factor, Ca – calcium, Cu – copper, Fe – iron, HC – healthy controls, hs-CRP – high sensitivity C-reactive protein, K – potassium, MDQ –Menstrual Distress Questionnaire, Na – sodium, PMS – premenstrual syndrome, PMTS – premenstrual tension syndrome, Mg – magnesium, PSST – premenstrual symptoms screening tool, RCT – Randomized Controlled Trial, Zn – Zinc

Importantly, in a prospective observation of three menstrual cycles and regular blood sampling, Chuong et al. noted that serum Zn levels are not only lower in PMS compared to healthy women, but also that a drop in serum Zn occurs in PMS subjects from the follicular to the luteal phase of the cycle. Conversely, serum Cu concentrations were higher in PMS than in controls during the luteal phase. In line with previous data, the Zn/Cu ratio was lower in PMS compared to healthy subjects during the luteal phase [52]. Some studies failed to detect significant differences in Zn levels during the luteal phase between PMS and controls [53–55]. One observed a higher luteal Zn/Cu ratio in the erythrocytes of PMS subjects compared to healthy controls [56]. This does not nullify the data on the role of the Zn/Cu ratio in PMD but rather indicates that the importance of their cycle-phase-dependent oscillations should be further examined in PMD subjects longitudinally, with more caution regarding the Zn/Cu ratio rather than crude Zn or Cu serum assessments.

### Zinc supplementation in PMD

A double-blind, randomized controlled trial (RCT) showed that 24 weeks of continuous supplementation with Zn sulfate (delivering 50 mg Zn) was more effective than a placebo in reducing premenstrual symptoms and improving school/work, family, and social functioning in women with PMS. Furthermore, the superiority of continuous Zn sulfate over placebo was significant after 1, 3, and 6 months of supplementation [57]. Additionally, a smaller RCT revealed that continuous 12-week Zn gluconate (30 mg Zn) supplementation was more effective than a placebo in alleviating the physical and psychological symptoms of PMS, while also increasing BDNF and TAC levels [58]. A large longitudinal 10-year cohort suggested that a high Zn intake of ≥ 25 mg/d (as measured by food frequency questionnaires) was linked to a lower risk of developing PMS. Still, only a trend in the significance of this association was observed [59]. Interestingly, a 3-month RCT showed that Zn sulfate 220 mg (50 mg Zn) supplemented only between the 16th and 2nd days of the menstrual cycle was superior to placebo in decreasing the severity of PMS over the subsequent 1, 2, and 3 months. It is worth noting that the superiority of Zn sulfate taken only between the 16th and 2nd days of the menstrual cycle over placebo in improving patients’ quality of life only became significant after 3 months [60]. In summary, data support the use of Zn in the treatment of PMS; it seems that at least 30 mg/d Zn supplementation for a period of at least a month (but preferably 2–3 months) is recommended, and treatment should continue throughout the menstrual cycle to achieve the best outcomes (Table 2).

## Copper

### Copper in premenstrual mood disorders

**Fig. 3:**
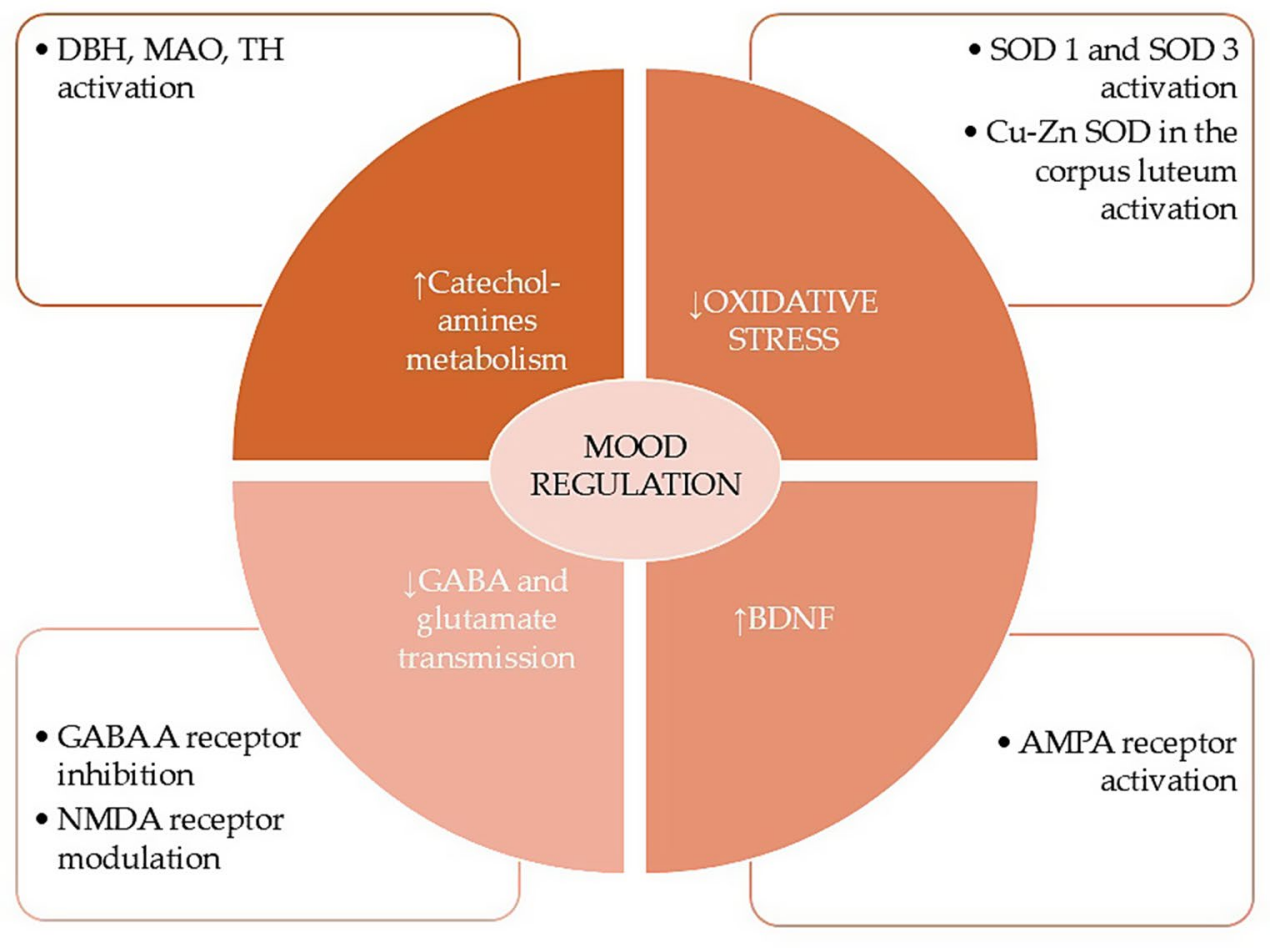
Copper’s impact on mood and the pathophysiology of premenstrual disorders

Cu is crucial to the activity of dopamine β-hydroxylase, monoamine oxidase, and tyrosine hydroxylase, which play a vital role in regulating monoaminergic transmission in the CNS [20]. Alterations in Cu levels or status often lead to psychopathological symptoms, particularly mood disorders [25]. Cu is also important for the proper function of superoxide dismutases (SOD) 1 and 3, and its deficiency might exacerbate oxidative stress [20], which has a significant role in affective disorders [61] and was implicated in PMD [62]. It is worth noting that specific antioxidant enzymes are present in the corpus luteum, including Cu–Zn SOD, which usually increases its activity in the luteal phase and eradicates superoxide radicals to stimulate progesterone production by the corpus luteum [63]. Cu might be necessary for maintaining appropriate progesterone levels and changes during the cycle. Cu appears to exert inhibitory effects on glutamatergic and GABAergic signaling, but promotes AMPAergic transmission, thereby enhancing synaptic plasticity and preventing excitotoxicity [20, 64]. Additionally, Cu inhibits GABA_A_ receptors. Interestingly, unlike Zn, Cu displays a similar affinity for all human GABA_A_ receptors [64] (Fig. 3).

Several studies have linked higher serum Cu concentrations to depressive symptoms [65]. In contrast, others showed that individuals who lack sufficient Cu intake in their diet are at higher depression risk [66] or did not detect differences in serum Cu levels between subjects with depression and healthy controls or any association between serum Cu and depression severity [67]. It was, however, indicated that Cu levels evolve along the disease progression in mood disorders [25]. Higher Cu concentrations might induce toxicity due to the production of free radicals [68], proteasome inhibition, and mitochondrial dysfunction, and they might induce cell death via Cu-specific cuproptosis pathways [69].

Copper is essential for the activity of several enzymes involved in monoaminergic transmission, specifically DBH, MAO, and TH. Cu also supports the functioning of SOD1 and 3. Additionally, Cu has been shown to inhibit glutamatergic and GABAergic signaling, while promoting AMPA-ergic transmission.

5-HT - serotonin, AMPA - α-amino-3-hydroxy-5-methyl-4-isoxazole propionic acid, BDNF - brain-derived neurotrophic factor, Cu - copper, DBH - dopamine β-hydroxylase, GABA - γ-amino butyric acid, MAO – monoamine oxidase, NMDA - N-methyl-D-aspartate, SOD - superoxide dismutase, TH - tyrosine hydroxylase, Zn – zinc.

### Serum copper levels and PMD

As mentioned above, increased serum Cu levels were observed in the luteal phase of the menstrual cycle in PMS patients vs. healthy controls (HC) [52]. In other studies, however, women with PMS or PMTS were reported to have similar or lower blood levels of Cu compared to HC [49, 56]. A lower mean plasma Zn/Cu ratio was also noted in PMTS women during the luteal phase compared to the follicular phase [49] (Table 2). Moreover, a small study found that PMS individuals had higher dietary Cu intake than controls, as measured using food diaries [70].

No studies on copper supplementation in PMD were found in the literature. Importantly, there is a lack of randomized controlled trials (RCTs) investigating the therapeutic role of copper in PMD. Such trials are needed to clarify whether Cu supplementation or intake limitation could play a beneficial role in managing PMD symptoms.

## Magnesium

### Magnesium impact on mood and PMD pathophysiology

**Fig. 4:**
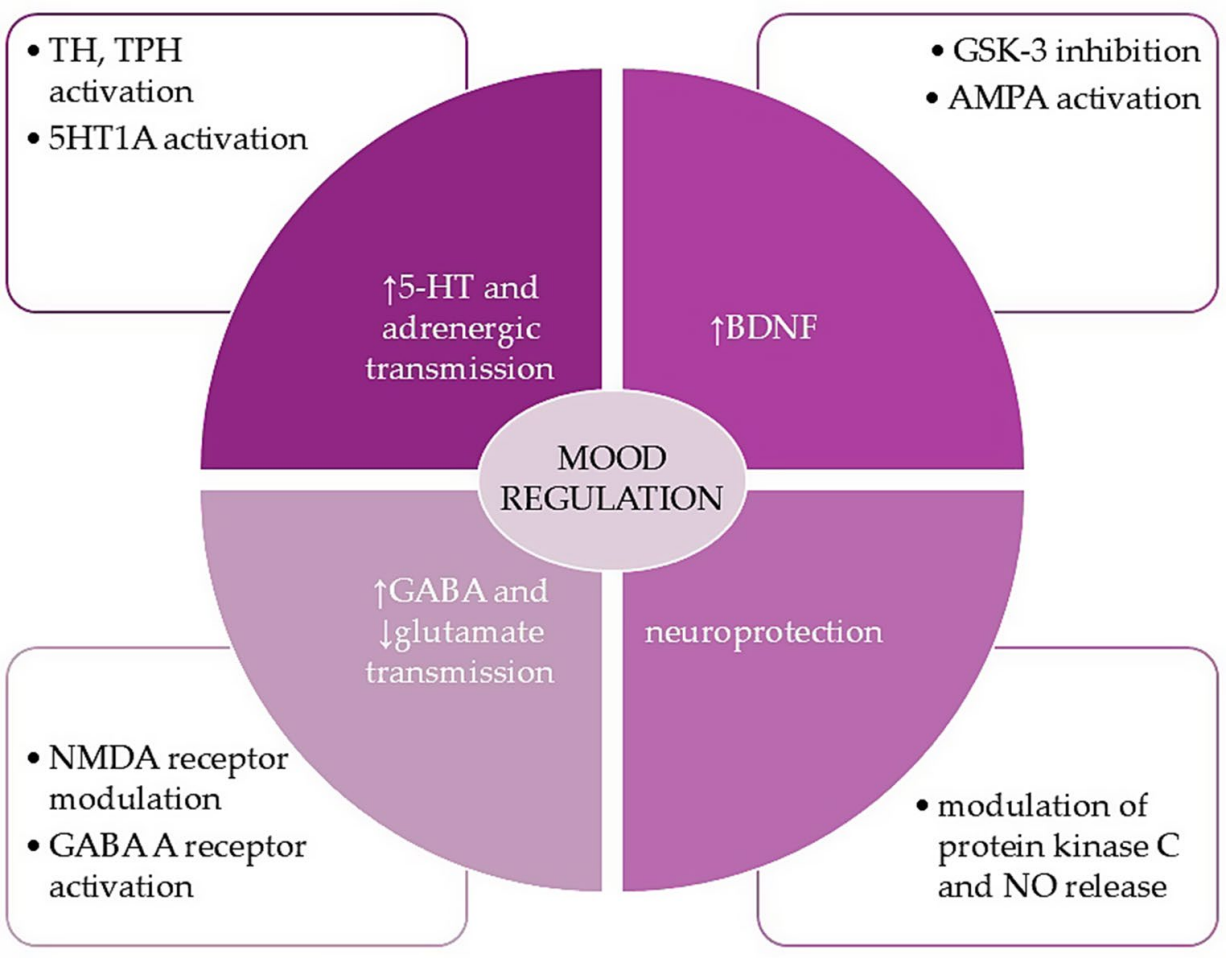
Magnesium’s impact on mood and the pathophysiology of premenstrual disorders

Mg is a cofactor for enzymes crucial to 5-HT and adrenergic signaling, specifically tyrosine and tryptophan hydroxylase [21]. It also directly promotes 5-HT transmission via 5-HT1A. Mg ions act antagonistically to calcium; they inhibit NMDA receptors by blocking the flow of calcium ions and promote the expression of the NMDA receptor GluN2B subunit, which is essential for ketamine’s antidepressant effects [42]. Additionally, Mg activates the GABA _A_ receptor, enhancing GABA-ergic signaling [21]. Furthermore, Mg contributes to antidepressant actions by increasing BDNF in the prefrontal cortex and activating Ca2+/calmodulin-dependent protein kinase II (CaMKII), which promotes AMPA signaling [24]. Moreover, Mg exerts antioxidant and neuroprotective activity through modulation of protein kinase C and nitric oxide (NO) release and promotes neurotrophic signaling via GSK-3 blockade [24] (Fig. 4). Mg deficiency is known to cause symptoms of depression, anxiety, and irritability [20] and to upregulate the HPA axis [71]. However, studies on serum Mg levels in affective disorders are inconsistent, with most indicating increased serum Mg levels during acute mood episodes [21, 72]. It has also been argued that a lower Ca/Mg ratio in the CNS and serum might be related to depression [24]. Additionally, it was reported that Mg and Zn levels are lower in the luteal phase compared to the follicular phase of the menstrual cycle, with Mg deficiency being significantly higher in the luteal phase [73], which might trigger PMD symptoms in vulnerable subjects.

Mg serves as a vital cofactor for key enzymes involved in serotonin (5-HT) and adrenergic signaling, specifically influencing the activity of TH and TPH. It enhances 5-HT transmission through the 5-HT1A receptor. Mg facilitates neurotrophic signaling through GSK-3 blockade and increases BDNF levels, promoting AMPA receptor signaling. It plays a role in neuroprotection by modulating protein kinase C and NO release. Mg inhibits NMDA receptors and increases GABA-ergic signaling via GABA_A_ receptor activation.

5-HT - serotonin, AMPA - α-amino-3-hydroxy-5-methyl-4-isoxazolepropionic acid, BDNF - brain-derived neurotrophic factor, GABA - γ-aminobutyric acid, NMDA - N-methyl-D-aspartate, SOD - superoxide dismutase, TH - tyrosine hydroxylase, GSK-3 - glycogen synthase kinase-3.

### Serum magnesium levels and PMD

Studies on Mg’s role in PMD are summarized in Table 3. One study noted lower monocyte Mg levels in PMS compared to controls, as well as lower Mg levels in erythrocytes in the subpopulation of PMS patients experiencing marked pain and behavioral changes [74]. Additionally, other research observed lower monocyte and red blood cell Mg levels in PMS compared to healthy women [10, 75, 76]. The erythrocyte Mg/Ca ratio was reported to be lower in PMTS/PMS compared to healthy participants [56, 77]. Some studies have found that women with PMTS have significantly lower plasma Mg levels compared to controls during both the follicular and luteal phases [49, 78–80], while others have reported similar serum Mg concentrations in women with PMTS/PMS/PMDD and healthy controls [53, 54, 81–84]. Furthermore, some data indicate luteal vs. follicular phase plasma Mg levels were lower in PMTS but not in healthy women [49]; however, other studies did not find significant differences in plasma Mg concentrations between phases or groups (PMS vs. controls) [75]. One cross-sectional study indicated that PMS subjects more frequently demonstrated serum Mg deficiency and reported lower dietary intake of both Mg and calcium, as assessed by a food recall questionnaire, compared to healthy women [85].

**Table 3:** Studies exploring the role of Mg in PMD

Study design	Population	Outcomes	References
Prospective observation, measurement of serum, monocyte, and erythrocyte Mg levels throughout the cycle (2–4 samples in the follicular, periovulatory, luteal, and premenstrual phases) PMS was assessed with a prospective administration of MDQ	18 women with PMS and 11 HC	No changes in serum or erythrocyte Mg levels throughout the cycle in PMS and HC Lower monocyte Mg in PMS vs. HC Lower erythrocyte Mg in PMS with increased pain and behavioral changes	[74]
Cross-sectional study, measurement of serum and erythrocyte Mg levels	26 women with PMTS and 9 HC	Lower erythrocyte (but not serum) Mg in PMS women vs. HC	[10, 77]
Prospective observation, measurement of serum, monocyte, and erythrocyte Mg levels 4 times during the cycle, 1 week apart in early and late follicular and luteal phases PMS symptoms were evaluated with a prospective 3-month daily monitoring symptom scale	26 women with PMS and 19 HC	Lower erythrocyte and monocyte Mg in PMS vs. HC at each sampling time	[75]
Cross-sectional study, serum and erythrocyte Mg and K levels assessed in the week before menses onset PMS was identified by interview and questionnaire	17 women with PMS	Normal serum Mg in all subjects, deficiency of erythrocyte Mg in 14 women	[76]
Cross-sectional study, assessment of Ca, Mg, Zn, Cu, and 14 other elements in blood samples (lack of data on the time of sample collection) and hair samples	46 women with PMS and 50 HC	No differences in Mg in PMS vs. HC blood	[56]
Cross-sectional study, measurement of serum Zn, Cu, Mg in the luteal phase (5 days before menses) and follicular phase (6 days after menses onset) Assessment of PMS with MDQ	40 women with PMTS and 20 HC	Lower mean plasma Mg levels in PMTS women vs. HC Lower mean plasma Zn/Cu ratio and Mg levels during luteal vs. follicular phase in PMTS women, but not in HC	[49]
Cross-sectional study, evaluation of serum Mg and vitamin D in the luteal phase	85 women with PMS	Lower Mg and vitamin D levels in PMS women vs. HC	[78]
Cross-sectional study, evaluation of serum Mg levels PMS assessed with PSS	Women with PMS and HC (data on sample size missing)	Lower serum Mg in PMS women vs. HC	[79]
Cross-sectional study, evaluation of serum Mg and Ca levels in pre- (15th -28th day) and post-menstrual (6th -14th day) phases of the cycle PMS was evaluated with a questionnaire based on ACOG criteria	50 women with PMS and 50 HC	Lower serum Mg in PMS women vs. HC in the premenstrual phase	[80]
Cross-sectional study, measurement of serum levels of vitamin D, Ca, Mg, Zn, Fe, K, Na taken 2 weeks after menses PMS assessed with PSST	115 women with PMS and 163 HC	No differences in Mg in PMS women vs. HC	[53]
Cross-sectional study, measurement of serum Zn, Mg, vitamin A, vitamin E, and activity of erythrocyte enzymes in the luteal phase (5 days prior to menses) and follicular phase (6 days after menses onset) PMTS diagnosed by a family doctor or a gynecologist	38 women with PMS (12 reported significant mood cyclicity) and 23 HC	No significant differences in Mg levels in the studied groups	[54]
10 years nested case-control study Mineral intake was assessed using food frequency questionnaires at the 1st, 5th, and 9th years of the study	1,057 women with PMS and 1,968 HC	Mg intake is unrelated to PMS risk	[59]
A 3-day food record during the perimenstrual phase was conducted to assess energy intake, diet composition, and consumption of macro- and micronutrients PMS was evaluated with DRSP	16 women with PMS and 14 HC	No association between Mg intake and PMS	[70]
Cross-sectional study, evaluations of serum and erythrocyte Mg in the mid-luteal phase	105 women with PMS and 50 HC	Lower erythrocyte Mg in PMS vs. HC Similar plasma Mg in both groups	[81]
Cross-sectional study, evaluations of serum Mg, pyridoxal phosphate, and erythrocyte aspartate aminotransferase stimulation, as well as food intake records in pre- and postmenstrual phases	9 women with PMS and 10 HC	Higher average Mg intake in HC vs. PMS in the premenstrual phase No differences in plasma Mg in the studied groups	[82]
Experimental study, evaluations of serum, erythrocyte, and monocyte Mg pre and post-intravenous Mg sulfate or placebo infusion in the luteal phase PMS was assessed with prospective daily symptom assessments for 3 cycles	17 women with PMDD and 14 HC	No differences in serum, erythrocyte, and monocyte Mg between PMDD and HC No impact of Mg sulfate infusion on mood in PMDD	[83]
RCT, cross-over, women first received vitamin B6 100 mg for 2 cycles, and later received placebo for 2 menstrual cycles, additionally serum Mg evaluations were done in the premenstrual phase of the 1st, 3rd and 5th cycle PMS was assessed with a scale ranking the symptom severity	34 women with PMTS and 10 HC	Serum Mg did not vary between PMTS and HC Vitamin B caused an increase in serum Mg Serum Mg was not correlated with PMTS severity	[84]
Cross-sectional study, evaluations of serum vitamin D, Ca, and Mg PMS assessed with the Utah PMS Calendar	31 women with PMS and 31 HC	Over a third of women with PMS had Mg deficiency Negative association between serum Mg and PMS risk	[85]
RCT, one group received Mg pyrrolidone carboxylic acid (360 mg Mg) and one group received placebo, trice daily for two months (from 15th day of the cycle to menses onset); later both groups received Mg for 2 months; measurements of serum, erythrocyte, polymorphonuclear cell and lymphocyte Mg levels in the premenstrual phase at baseline, after 2 and 4 months of the study PMS was prospectively assessed with MDQ	32 women with PMS	Treatment with Mg was superior to placebo in reducing the overall severity of PMS and the symptoms in the “negative affect” domain Mg supplementation increased polymorphonuclear cell and lymphocyte Mg levels	[86]
RCT, cross-over, one group first received Mg oxide (200 mg Mg) and one group received placebo, daily for 2 cycles; later the interventions were switched between the groups PMS was assessed with a daily symptoms scale recording	38 women with PMS	Mg was superior to placebo in reducing PMS “hydration” symptoms; the difference was significant in the 2nd cycle of intervention	[87]
RCT, cross-over each woman received consecutively all four interventions, each for one menstrual cycle: (1) MgO (200 mg Mg), (2) 50 mg vitamin B6, (3) MgO (200 mg Mg) and 50 mg vitamin B6, and (4) placebo PMS was assessed with the daily MHQ	44 women with PMS	Mg + vitamin B6 was effective in relieving PMS anxiety symptoms Mg or vitamin B6 monotherapy was comparable to placebo	[88]
Open-label, patented modified-release tablet 250 mg Mg taken from the 20th day of the cycle until menses for 3 months PMS assessed with modified MDQ and diaries	41 women with PMS	Significant decreases in PMS severity scores by over 33%	[89]
RCT, one group received Mg (250 mg Mg), one group received vitamin B6 (data on the dose not available), and one group received a placebo for 2 cycles PMS assessed with prospective 2-month menstrual diaries	126 women with PMS	Significant decreases in PMS severity scores, vitamin B6 was more effective than Mg and placebo, Mg was more effective than placebo	[90]

Ca – calcium, Cu – copper, DRSP – Daily Record of Severity of Problems, Fe – iron, HC – healthy controls, K – potassium, MDQ – Menstrual Distress Questionnaire, Na – sodium, PMS – premenstrual syndrome, PMTS – premenstrual tension syndrome, Mg – magnesium, MHQ – Menstrual Health Questionnaire, PMDD – premenstrual dysphoric disorder, PSST – Premenstrual Symptoms Scale, PSST – premenstrual symptoms screening tool, RCT – Randomized Controlled Trial, Zn – Zinc

### Magnesium supplementation in PMD

In a small RCT, women with PMS received Mg pyrrolidone acid (360 mg Mg) or placebo, dosed three times daily (from the 15th day of the cycle until menses) for two months. Next, all subjects received Mg pyrrolidone acid (360 mg Mg) for two additional months. The 2-month Mg pyrrolidone acid (360 mg Mg) supplementation was superior to placebo in reducing overall PMS severity and negative affect (but not pain). The 2-month Mg pyrrolidone acid (360 mg Mg, taken three times a day) treatment resulted in higher Mg levels in lymphocytes and polymorphonuclear cells (but not erythrocytes); however, there was no correlation between these parameters and PMS severity [86]. In another RCT, women received continuous MgO (200 mg Mg/d) or placebo for 2 months and were assessed for PMS symptoms. The results showed that continuous MgO (200 mg Mg/d) supplementation was more effective in reducing fluid retention symptoms of PMS than placebo, with the difference becoming apparent only in the second (but not first) month of treatment [87]. In a crossover RCT, each woman received all four interventions consecutively, each for one menstrual cycle: (1) MgO (200 mg Mg), (2) 50 mg vitamin B6, (3) MgO (200 mg Mg) plus 50 mg vitamin B6, and (4) placebo. The results indicated that Mg (as MgO heavy precipitate, 200 mg/d) and vitamin B6 (50 mg/d) were more effective than placebo in alleviating anxiety symptoms of PMS [88]. An open observation indicated that a 3-month treatment with 250 mg Mg/d modified-release tablets (dosed from the 20th day of the cycle until menses) significantly relieved all domains of premenstrual symptomatology by over 30% in subjects with PMS [89]. In a larger RCT, it was noted that continuous 2-month supplementation of Mg (250 mg/d) or vitamin B6 (data on the dose of vitamin B6 not presented) was superior to placebo in reducing the depression, anxiety, water retention, and somatic changes associated with premenstrual symptoms [90]. Mg supplementation was well-tolerated in all reported studies [86–90]. In short, the data support the use of Mg in treating PMS. It appears that continuous Mg supplementation at a dose of at least 200 mg/d (or supplementation only during the luteal phase with higher doses of 360 mg three times a day or 250 mg once a day in modified-release tablets) for at least 2 months is recommended (Table 3).

## Conclusions

The data presented above indicate that the cyclical hormonal fluctuations characteristic of the menstrual cycle, particularly in the luteal phase, have a significant impact on important neurobiological pathways associated with PMD, namely the functioning of the serotonergic, GABAergic systems, and the HPA axis. This neuroendocrine dysregulation co-occurs with other biological factors. Recently published data suggest that trace elements such as Zn, Cu, and Mg modulate the functions of the above-mentioned neurotransmitter systems. These micronutrients affect the same signaling pathways influenced by sex hormone fluctuations. They modulate receptor and enzyme functions and influence oxidative stress processes that mediate mood and stress responses. Understanding the interactions between Zn, Cu and Mg and hormone-sensitive neurobiological processes could help determine the mechanistic relationship between the menstrual cycle phase, micronutrient levels, and PMD symptoms.

In sum, fluctuations in Zn, Cu, and Mg levels during the follicular and luteal phases may modulate key neurotransmitter systems, such as serotonergic and GABAergic transmission, as well as the glutamate/GABA balance, which, according to the mechanisms described in the introduction, may contribute to the development of PMD symptoms.

There are several caveats to PMD and micronutrient research. Firstly, the length and complexity of the thorough diagnostic process (the daily recording of symptoms for two months) and the fact that some studies bypass this requirement, relying instead on retrospective questionnaires. This issue is also common in clinical practice, as many physicians omit the two-cycle diary recording recommended by gynecological and psychiatric guidelines [91]. Secondly, the diagnostic heterogeneity across studies: PMS, PMDD, and the historical construct PMTS were often used interchangeably, which limits comparability and highlights the need for future research applying standardized, prospective criteria. Thirdly, the fact that most micronutrient studies in PMD are based on very small samples and therefore require replication in larger groups. Moreover, these studies are characterized by varied designs, with non-homogeneous sampling windows, and lack the confirmation of ovulation or luteal status, thereby posing the risk of cycle phase misclassification, which could explain some of the null or discordant findings. The fourth caveat is that most of the available evidence comes from PMS research and needs confirmation in PMDD populations. The fifth caveat relates to funding transparency, which was only recently recognized as essential, while many of the studies included in this review were conducted 30–40 years ago.

The available studies indicate that continuous Zn supplementation throughout the menstrual cycle, at a dose of at least 30 mg/d for a duration of no less than one month (preferably 2–3 months), and continuous elemental Mg supplementation in a dose of at least 200 mg/d (or supplementation only in the luteal phase with higher doses of 360 mg three times a day or 250 mg once a day in modified-release tablet) for at least two months have a beneficial impact on PMD symptoms and are well-tolerated. However, it should be emphasized that the recommended supplementation doses are mainly based on studies with small sample sizes, and their effectiveness—as well as the broader role of Zn, Cu, and Mg in PMD—should be confirmed in large, well-designed clinical trials.

In addition, it is important to consider the safety of such supplementation therapies: the tolerable upper intake level (UL) for Zn is set at 40 mg/day in many jurisdictions, and multi-month use near this level may cause copper deficiency (hypocupremia) and anemia due to reduced copper absorption. Therefore, monitoring of Cu status (e.g., Zn: Cu ratio or serum Cu levels) may be advisable in individuals taking zinc at or near this threshold.

Moreover, caution should be exercised, as elemental Mg supplementation above 350 mg/day can lead to gastrointestinal adverse effects such as diarrhea.

Finally, neither Zn nor Mg should be considered substitutes for established first-line treatments, including SSRIs, cognitive behavioral therapy (CBT), or continuous combined oral contraceptives. Zn and Mg supplements could be considered low-cost, generally well-tolerated, and promising treatment options in PMD; however, larger, prospective trials (including RCTs in PMDD patients) with standardized outcomes are needed before any routine recommendation for Zn or Mg in PMD could be made. For the time being, Zn or Mg might be considered as potential adjunctive treatment options in patients who do not achieve satisfactory effects with SSRIs, CBT or contraceptives or as next-line treatments in patients who do not tolerate the first-line therapies.

Further research is needed to verify the initial findings on the role of Zn, Cu, and Mg in PMD using more robust methodologies. This should include large, well-controlled studies that follow standard diagnostic criteria, such as the recommended two-cycle symptom diary. It is also important to clearly distinguish between PMS and PMDD populations to ensure clinical relevance. Additionally, assessing micronutrient bioavailability across different phases of the menstrual cycle, along with the application of methods confirming ovulation/luteal status, could offer deeper insight into the role of Zn, Cu and Mg in PMD. Ultimately, this knowledge may support the development of targeted, evidence-based supplementation strategies for managing PMD.

## Data Availability

Data sharing not applicable to this article as no datasets were generated or analysed during the current study.

## Abbreviations

5-HT: Serotonin
AMPA: α-amino-3-hydroxy-5-methyl-4-isoxazolepropionic acid
BDNF: Brain-derived neurotrophic factor
CaMKII: Ca2+/calmodulin-dependent protein kinase II
CNS: Central nervous system
Cu: Copper
GABA: γ-aminobutyric acid
GnRH: Gonadotropin-releasing hormone
GSK-3: Glycogen synthase kinase-3
HC: Healthy controls
HPA: Hypothalamus-pituitary-adrenal
IPSCs: Inhibitory postsynaptic currents
Mg: Magnesium
Group I mGluRs: Glutamate receptors group I
Group II mGluRs: Glutamate receptors group II
NMDA: N-methyl-D-aspartate
NO: Nitric oxide
PMDD: Premenstrual dysphoric disorder
PMD: Premenstrual disorders
PMS: Premenstrual syndrome
PMTS: Premenstrual tension syndrome
RCT: Randomized controlled trial
SERT: Serotonin transporter
SOD: Superoxide dismutases
SSRIs: Selective serotonin reuptake inhibitors
TAC: Total antioxidant capacity
TPH: Tryptophan hydroxylase
Zn: Zinc
